# Malignant testicular tumors in children: A single institution’s 12-year experience

**DOI:** 10.1097/MD.0000000000029735

**Published:** 2022-07-22

**Authors:** Chia-Chi Chiu, Tang-Her Jaing, Jin-Yao Lai, Shih-Hsiang Chen, Tsung-Yen Chang, Chuen Hsueh, Yu-Chuan Wen, Pei-Kwei Tsay

**Affiliations:** a Department of Nursing, Chang Gung Memorial Hospital, Taiwan; b Division of Hematology and Oncology, Department of Pediatrics, Chang Gung Children’s Hospital, Chang Gung University, Taiwan; c Department of Pediatric Surgery, Chang Gung Children’s Hospital, Taiwan; d Department of Pathology, Chang Gung Memorial Hospital, Chang Gung University, Taiwan; e Department of Public Health and Center of Biostatistics, College of Medicine, Chang Gung University, Taiwan.

**Keywords:** histology, inguinal approach, orchiectomy, testicular tumor

## Abstract

Testicular neoplasms are not commonly found in children and are a formidable threat if treated inappropriately. However, there is no consensus regarding its management. This study aimed to create a holistic picture of the interprofessional team in the management of malignant testicular tumors.

Seventeen patients had mixed germ cell tumors, 15 had pure yolk sac tumors, 2 had immature teratomas, 2 had teratocarcinomas, and 1 had a sex cord stromal tumor. Five lesions were diagnosed as nongerm cell tumors: 2 embryonal rhabdomyosarcomas, 2 lymphomas, and 1 acute myeloid leukemia. At initial presentation, retroperitoneal (n = 2), bone marrow (n =1), and mediastinal (n = 1) metastases were identified in 4 (10%) patients. The operative interventions performed included radical inguinal orchiectomy (n = 5), scrotal orchiectomy (n = 31), and testicular biopsy or testis-sparing enucleation of the tumor (n = 6). Postoperatively, 18 patients received either adjuvant chemotherapy (n = 14) or chemoradiation (n = 5). Five patients with mixed germ cell tumors (n = 2), group IV paratesticular rhabdomyosarcoma (n = 2), and acute myeloid leukemia with myeloid sarcoma (n =1) died of disease progression. Thirty-six patients remained alive and disease-free at the last visit.

Malignant testicular tumors in children deserve proper diagnostic support from a therapeutic perspective. Any concern or suspicion of a testicular tumor warrants an inguinal approach to avoid scrotal violation.

## 1. Introduction

Testicular tumors are relatively rare in children, accounting for only 1% of all solid tumors diagnosed.^[1]^ Pediatric prepubertal testicular tumors are dramatically different from adult neoplasms. Germ cell tumors account for only 60% to 77% of testicular tumors in children but account for 95% of testicular tumors in adults.^[2]^ The proportion of benign testicular tumors in children is significantly higher than that in adults, affecting the evolution of the diagnosis and treatment for pediatric testicular tumors.^[3,4]^ These results complement pediatric research data, suggesting that prepubertal tumors should be treated differently from adult testicular tumors.

We highlight the study not only for the clinical presentation, diagnosis, and management of these children but also for heightening the clinician’s awareness of this rare group of tumors.

## 2. Materials and Methods

### 2.1. Data source

This institutional-based retrospective cohort study evaluated the clinical presentation, histopathologic diagnosis, treatment, and outcome of 42 boys with malignant neoplasms of the testis treated between May 2006 and November 2019.

We retrospectively investigated the outcomes in 42 boys aged 0 to 19 years presenting with testicular masses. Patients were reviewed and retrospectively analyzed based on their age at admission, presenting complaints, clinical and radiological findings, tumor markers, management, and follow-up. Pathology was centrally reviewed (C.H.) in patients with a tissue biopsy, but confirmation by a central review was not required before enrollment. This retrospective study was approved by the institutional review board of Chang Gung Memorial Hospital. Their parents or legal guardians provided written informed consent through an opt-out method on our hospital website, following the ethical principles for medical research involving human subjects in Taiwan.

The evaluation included age at presentation, medical history, clinical characteristics, diagnostic procedures, treatment methods, histopathological findings, and outcomes for every patient. All patients underwent color Doppler ultrasonography of the testis and abdomen, plain radiography of the thorax, and serum alpha-fetoprotein (AFP) levels before surgery. AFP levels are usually very high in newborns and do not decrease to normal adult levels until 8 months. Thus, AFP levels in infants were evaluated according to the reference ranges.^[5,6]^

All 42 tumors were treated surgically. The groin was explored using an inguinal incision. The testicle was gently grasped and exteriorized, and the spermatic cord and vessels within the tunica vaginalis were clamped. The resected tumor mass was sent for frozen-section examination (FSE). Testis-sparing surgery was performed on patients with nongerm cell tumors. However, radical orchiectomy was performed if the tumor had replaced the entire testis. All tumors were histologically analyzed. The optimal follow-up protocol included regular control visits with physical examinations, AFP measurements, and testicular sonography. Adjuvant chemotherapy was administered depending on the histopathology and TNM criteria for tumor (T), node (N), and metastases (M) classification. If nongerm cell tumors are assessed accurately on FSE, testis-sparing surgery could be an alternative treatment modality.

### 2.2. Statistical analysis

Statistical analyses and calculations were performed using SPSS version 20.0 (IBM, USA). A Kaplan–Meier survival curve was constructed to determine the survival rate and time. Differences between groups were compared using the log-rank test. Adjusted analysis was not performed due to the low numbers of patients in the compared groups.

## 3. Results

A summary of the clinical data for the 42 patients is presented in Table 1. All patients were diagnosed as children: 7 (16.7%) were ≤1 year, 13 (31.0%) were >1 year but ≤3 years, 3 (2.4%) were >3 years but ≤10 years, and 19 (45.2%) were >10 years but ≤18 years. The median age at initial presentation in this cohort was 36 months (range, 0.3–18 years). The most common symptom was a painless scrotal mass or swelling in 95.2% (40/42) of the cases. There were 22 right-sided and 18 left-sided tumors. Of the tumors, 39 were malignant, and 3 were benign (2 immature teratomas and 1 sex cord-stromal tumor).

**Table 1:** Clinical characteristics and management of testicular tumors.

**Pathology**	**No (%**)	**Median age at presentation** **(mo**)	**Affected side**		**Initial surgery**		**Median follow-up (mo**)
			**Left**	**Right**	**Testicular-sparing surgery**	**Radical inguinal orchiectomy**	
Mixed germ cell tumor	17	40	8	9	1	16	55
Pure yolk sac tumor	15	36	6	9	0	15	46
Teratocarcinoma	2	5	2	0	0	2	87
Immature teratoma	2	5	0	2	0	2	132
Sex cord stroma tumor	1	2	0	1	0	1	79
Nongerm cell tumor							
Embryonal rhabdomyosarcoma	2	5	1	1	2	0	12
Lymphoma	2	5	1	1	2	0	27
Acute myeloid leukemia	1	2	0	1	1	0	13

Doppler scrotal ultrasound was used in all patients, and testicular and paratesticular lesions were detected. Radiological assessment by computed tomography of the abdomen and pelvis was performed in 31 patients with high AFP and human chorionic gonadotrophin levels. Forty-two patients with a median age of 3.2 years were treated for malignant testicular tumors. All patients presented with a testicular mass. Seventeen patients had mixed germ cell tumors, 15 had pure yolk sac tumors, 2 had immature teratomas, 2 had teratocarcinoma, and 1 had a sex cord stromal tumor. Five lesions were diagnosed as nongerm cell tumors: 2 embryonal rhabdomyosarcomas (spindle cell variant), 2 lymphomas, and 1 acute myeloid leukemia. At initial presentation, retroperitoneal (n = 2), bone marrow (n =1), and mediastinal (n = 1) metastases were recorded in 4 (10%) patients. Operative procedures included radical inguinal orchiectomy (n = 5), scrotal orchiectomy (n = 31), and testicular biopsy or testis-sparing enucleation of the tumor (n = 6).

**Figure 1.**
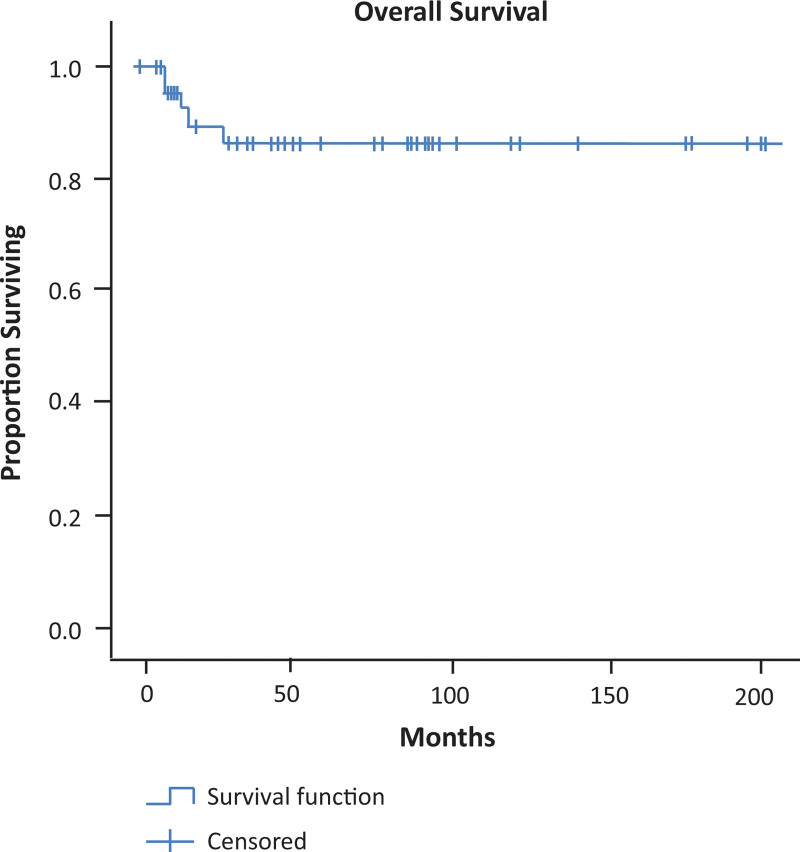
The overall survival rate in all patients from the time of first therapy in our institution (months)

Postoperatively, 18 patients received either adjuvant chemotherapy (n = 14) or chemoradiation (n = 5). Five patients with mixed germ cell tumors (n = 2), group IV paratesticular rhabdomyosarcoma (n = 2), and acute myeloid leukemia with myeloid sarcoma (n =1) died of disease progression. One patient with yolk sac tumor was alive despite progressive disease at 25 months of follow-up. One patient was diagnosed with growing teratoma syndrome 10 months after treatment for mixed germ cell tumors. All the remaining patients were alive and disease-free at their last outpatient appointment. The median follow-up time was 54 months (2–205 months; 95% confidence interval, 3.47–6.93) months: surgery. Considering all stages, the 1- and 5-year overall survival rates for all patients were 91.8 and 85.8%, respectively (Fig. 1).

## 4. Discussion

The population of patients with malignant testicular tumors is heterogeneous. Benign and malignant tumors are frequent in prepubertal and pubertal boys, respectively. Testicular-sparing surgery is the preferred treatment for benign tumors. Radical inguinal orchiectomy is indicated when a malignant tumor is identified.^[2,7]^ Their rarity, centralized pathology and treatment, and national collaborative clinical trials have helped establish this group’s optimum management of malignant tumors.^[8]^ Retroperitoneal lymph node dissection and radiation therapy play minimal roles,^[9]^ encouraging us to pursue this treatment further. Childhood testicular tumors deserve special diagnostic attention from a therapeutic perspective. Testicular tumors should be considered malignant until otherwise proven.^[10]^ In suspected testicular tumors, the inguinal approach is preferred to the scrotal approach for scrotal violation. Furthermore, FSE may improve the diagnostic accuracy, favoring an organ-sparing approach, especially in doubtful cases.^[11]^

The treatment of testicular yolk sac tumors is dependent on tumor stage and patient age. Early-stage tumors fare better than late-stage tumors do. Resection and chemotherapy with or without retroperitoneal lymph node dissection are often used in children with elevated or rising AFP levels and/or retroperitoneal lymphadenopathy.^[12]^ The differences in outcomes with increasing age are probably related to changes in disease biology and patient characteristics. Under this approach, pure immature teratomas were excluded from recent studies on pediatric malignant germ cell tumors.^[13]^ In the ovary, mature teratomas are considered benign and immature teratomas are considered malignant, unlike testis.^[14]^

Our study has some limitations. First, the sample size was small because of the rarity of the disease and its single-institutional nature. Second, there may have been an inherent selection bias. Genetic and molecular markers were not taken into account. The patients were referred to a tertiary hospital. This could have increased the prevalence of severe underlying pathologies in our study. Third, this study was a retrospective analysis with inherent selection bias. The random error arises due to variation between samples that might be drawn in a study and can be reduced by increasing the sample size.^[15]^ Because our data were obtained from 1 ethnicity, the direct application of these data to other races might be inappropriate.

## 5. Conclusion

Early diagnosis is vital for effective disease management. A solid scrotal mass should be regarded as potentially malignant until otherwise proven. Treatment strategies for testicular cancer require specialized input. Given the remaining uncertainties regarding malignant testicular tumors in children, further research on this population is warranted to address unanswered questions.

## Author contributions

Conceptulaization: Chia-Chi Chiu, Tang-Her Jaing

Data curation: Yu-Chuan Wen

Formal analysis: Pei-Kwei Tsay

Investigation: Tang-Her Jaing, Shih-Hsiang Chen

Methodology: Tang-Her Jaing, Jih-Yao Lai, Shih-Hsiang Chen

Supervision: Tsung-Yen Chang

Validation: Pei-Kwei Tsay, Chuan Hsueh

Writing – original draft: Yu-Chuan Wen, Chia-Chi Chiu

Writing – review & editing: Tang-Her Jaing
